# Acacetin alleviates neuroinflammation and oxidative stress injury via the Nrf2/HO-1 pathway in a mouse model of spinal cord injury

**DOI:** 10.1515/tnsci-2022-0266

**Published:** 2022-12-21

**Authors:** Xin Zhang, Lijun Xu, Xiang Chen, Xianjie Zhou, Lanhua Cao

**Affiliations:** Department of Orthopedic, Huangshi Central Hospital, Affiliated Hospital of Hubei Polytechnic University, Edong Healthcare Group, No. 141 Tianjin Road, Huangshi 435000, Hubei, China; Department of Radiology, Huangshi Central Hospital, Affiliated Hospital of Hubei Polytechnic University, Edong Healthcare Group, Huangshi 435000, Hubei, China

**Keywords:** spinal cord injury, acacetin, HO-1, Nrf2

## Abstract

Spinal cord injury (SCI) is a severe central nervous system disease, which may cause serious locomotor deficit. Acacetin is a flavone that possesses antioxidant and anti-inflammatory effects in different human diseases. The main purpose of this study was to explore whether acacetin ameliorates SCI in mice. A model of SCI was established in C57BL/6 mice. The Basso Mouse Scale (BMS) score, BMS subscore, mechanical hypersensitivity, and thermal hypersensitivity of mice were tested for determining the motor function. Immunofluorescence staining was utilized to detect NeuN, GFAP, and Iba-1 levels in spinal cord tissues. ELISA was utilized to assess the contents of proinflammatory factors such as interleukin (IL)-1β, IL-18, and tumor necrosis factor-alpha (TNF-α) in spinal cord tissues. The levels of oxidative stress markers, reactive oxygen species, thiobarbituric acid-reactive substances, superoxide dismutase, catalase, glutathione peroxidase, and glutathione were detected using their corresponding kits. Western blot was employed for estimating the levels of heme oxygenase 1 (HO-1), nuclear factor E2-related factor 2 (Nrf2), and Kelch-like ECH-associated protein 1 (Keap-1). In this study, acacetin treatment recovered the motor function in SCI mice. Acacetin improved neuron integrity and repressed glial cell activation in the spinal cord tissues of SCI mice. Furthermore, acacetin administration reduced the SCI-induced high concentrations of IL-1β, IL-18, and TNF-α, as well as inhibited oxidative stress in SCI mice. Moreover, acacetin activated HO-1/Nrf2 pathway in SCI mice. The neuroprotective effects of acacetin against SCI were reversed by Nrf2 inhibitor. Overall, acacetin alleviated neuroinflammation and oxidative stress injury by activating the Nrf2/HO-1 signaling pathway in the mouse models of SCI.

## 1 Introduction

Spinal cord injury (SCI) is a severe central nervous system (CNS) disease [1]. Mechanical compression directly damages the spinal cord, resulting in dysfunction of the limb function below the injured segment, and even paralysis in severe cases [2,3]. SCI has the characteristics of high incidence, high disability rate, high cost, low mortality, and young age [4]. It not only brings serious physical and psychological damage to the patients themselves, but also causes a huge economic burden to the whole society [5]. The pathological changes of SCI caused by external violence include primary injury and secondary injury [6]. Primary injury refers to the destruction of local tissue structure and cell death caused by external injury factors [7]. Secondary injury refers to the apoptosis and functional damage caused by oxidative stress and inflammatory reaction in the lesion site [8]. Primary injury can be treated in time by surgical intervention, while secondary injury will persist and affect the recovery of spinal cord function [9].

Neuroinflammation plays a key role in the secondary injury of SCI [10–12]. Microglia are the main innate immune cells of CNS and play vital roles in neuroinflammation [13]. After the occurrence of SCI, microglia are activated, and they induce the upregulation of proinflammatory factors [14]. Excessive activation of microglia leads to the overproduction of proinflammatory factors (such as interleukin [IL]-18, IL-1β, and tumor necrosis factor-alpha [TNF-α]) and reactive oxygen species (ROS), which aggravate inflammation and injury [14,15]. Additionally, more and more studies have confirmed the participation of oxidative stress in SCI pathogenesis [16]. Oxidative stress is the cell damage caused by excessive production of ROS [17]. Inhibiting the occurrence of oxidative stress can effectively repress the development of neurodegenerative diseases [18,19]. The pathological basis of secondary SCI caused by external violence is the necrosis of spinal cord neurons, the destruction of synaptic structure, and the loss of function [20]. Injury mediated by local ischemia and hypoxia produces extensive oxygen free radicals, and induce oxidative stress and neuronal apoptosis, which is one of the main pathological processes aggravating nerve injury [21,22]. The regeneration ability of neurons in the spinal cord tissue is weak, and it is difficult to repair after injury [23]. Therefore, antioxidant stress is an important target for the treatment of SCI and the improvement of neurological function.

Acacetin (5,7-dihydroxy-4ʹ-methoxyflavone) is a flavone present in plants including chrysanthemum and safflower [24,25]. It has been confirmed to possess assorted biological activities including anti-inflammation, antioxidant, and anticancer [24,26]. The neuroprotective effects of acacetin have been widely reported [27]. For example, acacetin suppresses glutamate release and prevents neurotoxicity of rats [28]. Acacetin represses neuronal apoptosis in Parkinson’s disease [29]. Acacetin protects dopaminergic cells against MPTP-induced neuroinflammation [30]. Moreover, acacetin inhibits microglial activation in lipopolysaccharide (LPS)-stimulated BV-2 cells and LPS-induced neuroinflammation mouse models, suggesting that acacetin could function as a promising therapeutic agent for brain disorder related to neuroinflammation [31]. Nevertheless, the function of acacetin in SCI remains unclear.

In this study, we aimed to investigate the specific function of acacetin in SCI. We hypothesized that acacetin could alleviate neuroinflammation and oxidative stress injury in SCI, which may provide a potential drug candidate for SCI treatment.

## 2 Materials and methods

### 2.1 Animal and study design

Female C57BL/6J mice (20–25 g, 8-week old) were obtained from Vital River Co. Ltd (Beijing, China).

The mice were kept at 22–24°C for 1 week of adaptive feeding. They had free access to food and drink. The animal experiment was divided into two parts. In part 1, mice were randomly allocated into (1) sham + normal saline (NS) group, (2) sham + acacetin group, (3) SCI + NS group, and (4) SCI + acacetin group (*n* = 8 each group). Acacetin (15, 30, and 50 mg/kg) was dissolved in NS containing 5% dimethylsulfoxide (DMSO) and administered intraperitoneally (i.p.) daily for 42 consecutive days. Mice in the sham + NS and SCI + NS groups were injected i.p. with equivalent volume of NS containing 5% DMSO as vehicle, and mice in the Sham + acacetin and SCI + acacetin groups received i.p. injection of 15, 30, and 50 mg/kg acacetin at 30 min post-surgery and then once a day for 42 successive days, respectively. In part 2, mice were randomized into the sham, SCI, SCI + acacetin (15 mg/kg), and SCI + acacetin (15 mg/kg) + ML385 (30 mg/kg, inhibitor of nuclear factor E2-related factor 2 [Nrf2]) (*n* = 8 each group). ML385 was dissolved in 100% DMSO to prepare a stock solution and then diluted it into 5% DMSO solution with NS (vehicle) before being used [32]. Acacetin (15 mg/kg) was dissolved in NS containing 5% DMSO. Mice in the sham and SCI groups were injected i.p. with equivalent volume NS containing 5% DMSO, mice in the SCI + acacetin group were injected i.p. with 15 mg/kg acacetin at 30 min post-surgery once a day, and mice in the SCI + acacetin + ML385 group received i.p. injection of 30 mg/kg ML385 at 30 min before administration of 15 mg/kg acacetin at 30 min post-surgery and then once a day, respectively, for 42 consecutive days.


**Ethical approval:** The research related to animals’ use has been complied with all the relevant national regulations and institutional policies for the care and use of animals. The animal study was approved by the Ethics Committee of Huangshi Central Hospital (Hubei, China).

### 2.2 SCI model

Mice were deeply anesthetized with sodium pentobarbital (40 mg/kg); then, laminectomy was performed at the level of T9–T10 of the mouse spine, completely exposing the dorsal side of the spinal cord. Next, a mouse spinal cord impactor (RWD, Cat#Model III, USA, 2 mm diameter, 12.5 g, 20 mm in height) was used to cause a moderate contusion on the surface of the spinal cord at T9–T10 (a more severe contusion often results in obvious mortality). The mice in the sham operation experienced the same procedure, excluding the spinal cord contusion. After the operation, the surface of the spinal cord was hemorrhaged, and the mice had straight legs and drooping tails, which indicates that the mouse model of moderate SCI was constructed successfully. The muscles, fascia, and skin of the mice were sutured layer-by-layer and disinfected with iodophor. The bladder was emptied manually every day for the next few days until the mice regained spontaneous urination function.

### 2.3 Detection of locomotion function, mechanical, and thermal hypersensitivity

A locomotion function assessment was conducted utilizing the Basso Mouse Scale (BMS) score and the BMS subscore for 42 days after SCI [33]. Mice were freely moved for 5 min. Then, the locomotor function of hind limb movement in mice was scored according to BMS with a score of 0–9 points (0 points mean complete paralysis and 9 points mean completely normal). The well-trained investigators were blinded to score them on the BMS. The BMS scores of the left and right hind limbs were calculated and averaged to get a single value for each test of each mouse.

Mechanical allodynia was detected by hind paw withdrawal [34]. The withdrawal threshold was calculated as the average of the left and right hind paws to get the single value for each mouse. Thermal hyperalgesia was estimated via detecting hind paw withdrawal latency responding to a stimulation of radiant heat managed via a plantar test analgesia meter (IITC, Life Science, USA) [35]. To prevent tissue injury, a cut-off of 20 s was utilized. The average of three trials was utilized for reporting the final withdrawal latency.

### 2.4 Hematoxylin–eosin (H&E) staining

Mice were anesthetized at 14 days following SCI and then perfused with NS and 4% paraformaldehyde through the heart. The lesioned spinal cord was about 4 mm in length. After perfusion, the spinal cord containing the epicenter of injury was dissected out from each mouse (approximately 10 mm) and fixed in 4% paraformaldehyde for 24 h and embedded in paraffin for transverse sectioning. Transverse sections (4 µm thickness) were prepared on poly-l-lysine-coated slides for histopathological analysis by H&E according to the manufacturers’ instructions. Images were captured under a light microscope (Nikon, Tokyo, Japan).

### 2.5 Immunofluorescence staining

Spinal cord tissue sections were fixed with 4% paraformaldehyde for 15 min, permeabilized with 0.1% Triton X-100 for another 15 min, and then blocked with 10% normal goat serum for 1 h. The washed sections were incubated with specific primary antibodies including NeuN (1:300, ab177487; Abcam, USA), GFAP (1:50, ab4648; Abcam), and Iba-1 (1:500, ab178846; Abcam) at 4°C overnight. On the next day, the primary antibody was washed off with 1× PBS, and the corresponding fluorescent secondary antibody (Alexa Fluor 594 goat anti-mouse IgG or Alexa Fluor 488 goat anti-rabbit IgG; 1:250, Thermo Fisher Science) and the spinal tissues were subsequently incubated together for 1 h at room temperature. Next, the cell nuclei were stained with 4′6-diamino-2-phenylindole (Invitrogen, Carlsbad, CA, USA) solution. Finally, a fluorescence microscope (Olympus, Tokyo, Japan) was used to observe and image the spinal cord tissue, and ImageJ2x software was utilized to analyze the average optical density or count the fluorescence-positive cells in randomly selected images in different tissues.

### 2.6 Western blot

The spinal cord tissues were lysed by RIPA buffer (Thermo Fisher Scientific, Waltham, MA, USA), and the protein concentrations were tested by BCA kit (Beyotime, Shanghai, China). Proteins were isolated by 10% SDS‐PAGE and transferred onto PVDF membranes (Millipore, Billerica, MA, USA), which were then blockaded with 5% skim milk. After that, membranes were cultured with primary antibodies at 4°C overnight. Then, the membranes were rinsed with tris-buffered saline-tween-20 and cultured with the secondary antibody (1:2,000, ab6789; Abcam) for 2 h. The membranes were visualized by enhanced chemiluminescence luminescent liquid (Advansta, Menlo Park, CA, USA). The relative density of protein bands was analyzed by ImageJ (v1.8.0; National Institutes of Health). The primary antibodies used are as follows: NeuN (1:1,000, ab177487; Abcam), GFAP (1:5,000, ab207165; Abcam), Iba-1 (1:500, ab178846; Abcam), heme oxygenase 1 (HO-1; 1:2,000, ab52947; Abcam), Nrf-2 (1:1,000, #12721, Cell Signaling Technology, USA), Kelch-like ECH-associated protein 1 (Keap-1; 1:2,000, ab227828; Abcam), and GAPDH (1:10,000, ab181602; Abcam) served as loading control.

### 2.7 Biochemical analysis

Spinal cord segments containing the injury epicenter and surrounding uninjured tissues were dissociated and homogenized. The homogenates were centrifuged at 4°C at 12,000*g* for 15 min, and supernatants were collected and evaluated for ELISA analysis using interleukin 1β (IL-1β), tumor necrotic factor-α (TNF-α), and IL-18 commercial kits following the introduction of kits (X-Y Biotechnology, Shanghai, China).

Commercial kits were used for determining lipid peroxidation (thiobarbituric acid-reactive substances [TBARS]; Cayman Chemical, USA), ROS, catalase (CAT), glutathione peroxidase (GPX), glutathione (GSH), and superoxide dismutase (SOD) activities (Beyotime) following the manufacturer’s instructions.

### 2.8 RT-qPCR

The total RNA was extracted from spinal cord tissue using 1 mL of TRIzol (Invitrogen, Carlsbad, CA, USA). Then, RNA was used for reverse transcription to synthesize cDNA using Reverse Transcription Kit (Qiagen, Hilden, Germany). The qPCR was conducted utilizing SYBR Green (Promega, Fitchburg, WI, USA) on StepOnePlus Real-Time PCR System (Applied Biosystems, USA). The expression of TNF-α, IL-1β, and IL-18 was calculated utilizing the 2^−ΔΔCt^ method and normalized to GAPDH. The sequences of primers used are as follows:

TNF-α forward, 5′-GCCTCTTCTCATTCCTGCTTG-3′;

TNF-α reverse, 5′-CTGATGAGAGGGAGGCCATT-3′;

IL-1β forward, 5′-TGGCAACTGTTCCTG-3′;

IL-1β reverse, 5′-GGAAGCAGCCCTTCATCTTT-3′;

IL-18 forward, 5′-CCTTTGAGGAAATGGATCCAC-3′;

IL-18 reverse, 5′-GTCCTGGAACACGTTTCTG-3′;

GAPDH forward, 5′-ACTCTTCCACCTTCGATGC-3′;

GAPDH reverse, 5′-CCGTATTCATTGTCATACCAGG-3′.

### 2.9 Statistical analyses

Data are displayed as mean ± SD from three individual repeats. GraphPad Prism 8 software (GraphPad Software, Inc., La Jolla, CA, USA) was applied for statistical analysis. Data were analyzed by one-way ANOVA followed by Tukey’s *post hoc* analysis. *p* < 0.05 represented statistical significance.

## 3 Results

### 3.1 Acacetin attenuates motor function of SCI mice

**Figure 1:**
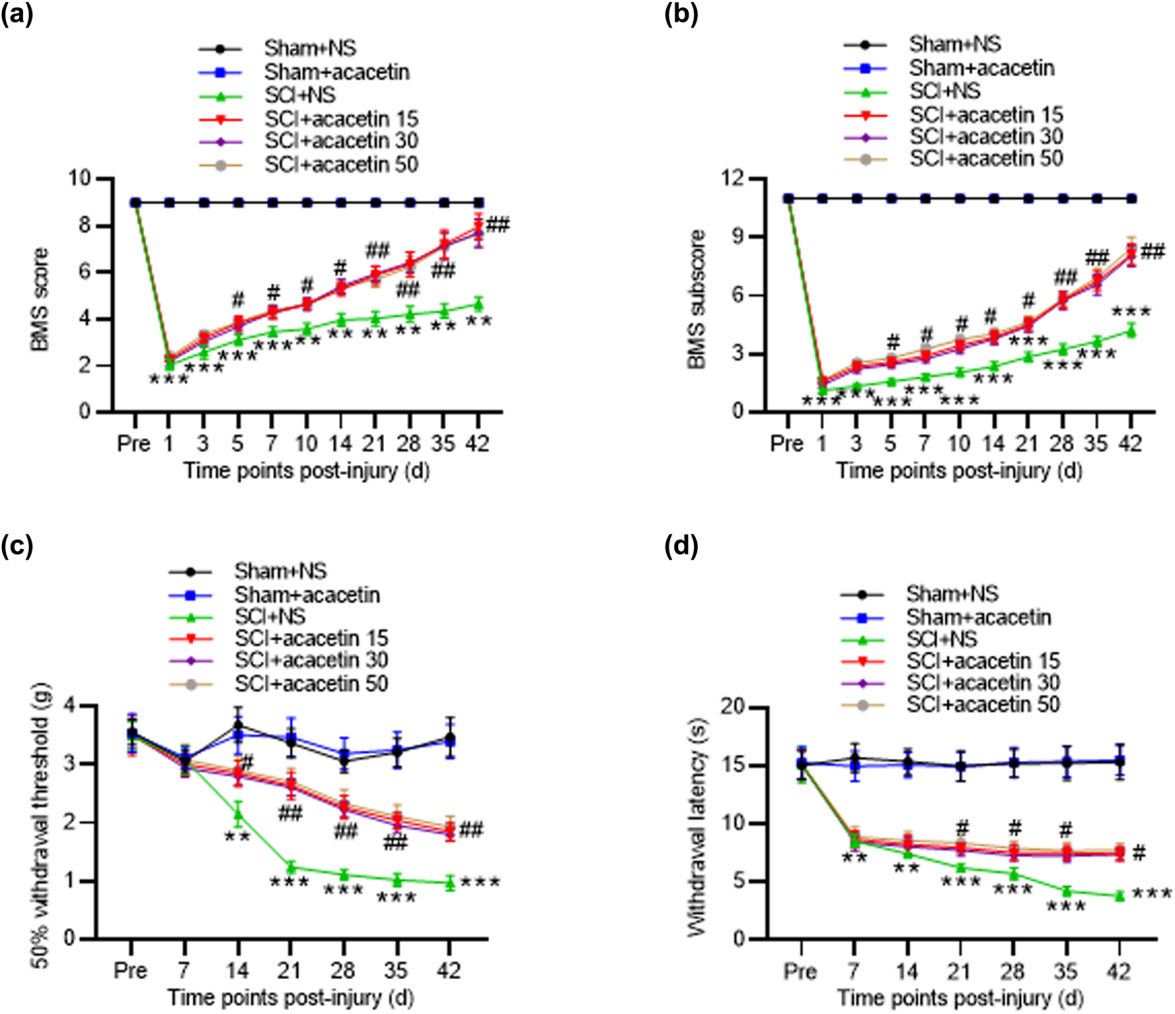
Acacetin attenuates motor function of SCI mice. (a and b) BMS score and BMS subscores in mice of different groups (sham + NS group, sham + acacetin group, SCI + NS group, and SCI + acacetin group) were evaluated. (c and d) Mechanical hypersensitivity and thermal hypersensitivity of mice in different groups were detected using withdrawal threshold and withdrawal latency. *n* = 8 each group. **p < 0.01, ***p < 0.001 (compared with the sham + NS group); ^#^ *p* < 0.05, ^##^ *p* < 0.01 (compared with the SCI + NS group).

To explore the biological roles of acacetin in SCI, we established a mouse model of SCI and performed a series of assays. According to the results, we discovered that the sham-operated mice treated with NS or acacetin showed no dyskinesia within 42 days. BMS score of mice treated with acacetin was higher than that of mice treated with NS from Days 5 to 42 after SCI (Figure 1a). The BMS subscore of mice in the sham + NS and sham + acacetin groups had no change from the beginning to the end of 42 days, while SCI mice treated with acacetin showed better motor function than SCI mice treated with NS (Figure 1b). Then, the withdrawal thresholds in sham + NS group and sham + acacetin group did not display significant change within 42 days. The withdrawal thresholds of mice in SCI + acacetin group were higher than those in SCI + NS group (Figure 1c). Similarly, the withdrawal latency in acacetin-treated SCI mice was significantly longer than that in NS-treated SCI mice (Figure 1d). Additionally, we found that BMS score and mechanical and thermal hypersensitivity had no significant change among SCI + acacetin (15 mg/kg), SCI + acacetin (30 mg/kg), and SCI + acacetin (50 mg/kg) groups (Figure 1a–d), suggesting that the effect of acacetin may not be concentration-dependent. We used 15 mg/kg acacetin in the subsequent assays.

### 3.2 Acacetin represses glial cell activation after SCI

**Figure 2:**
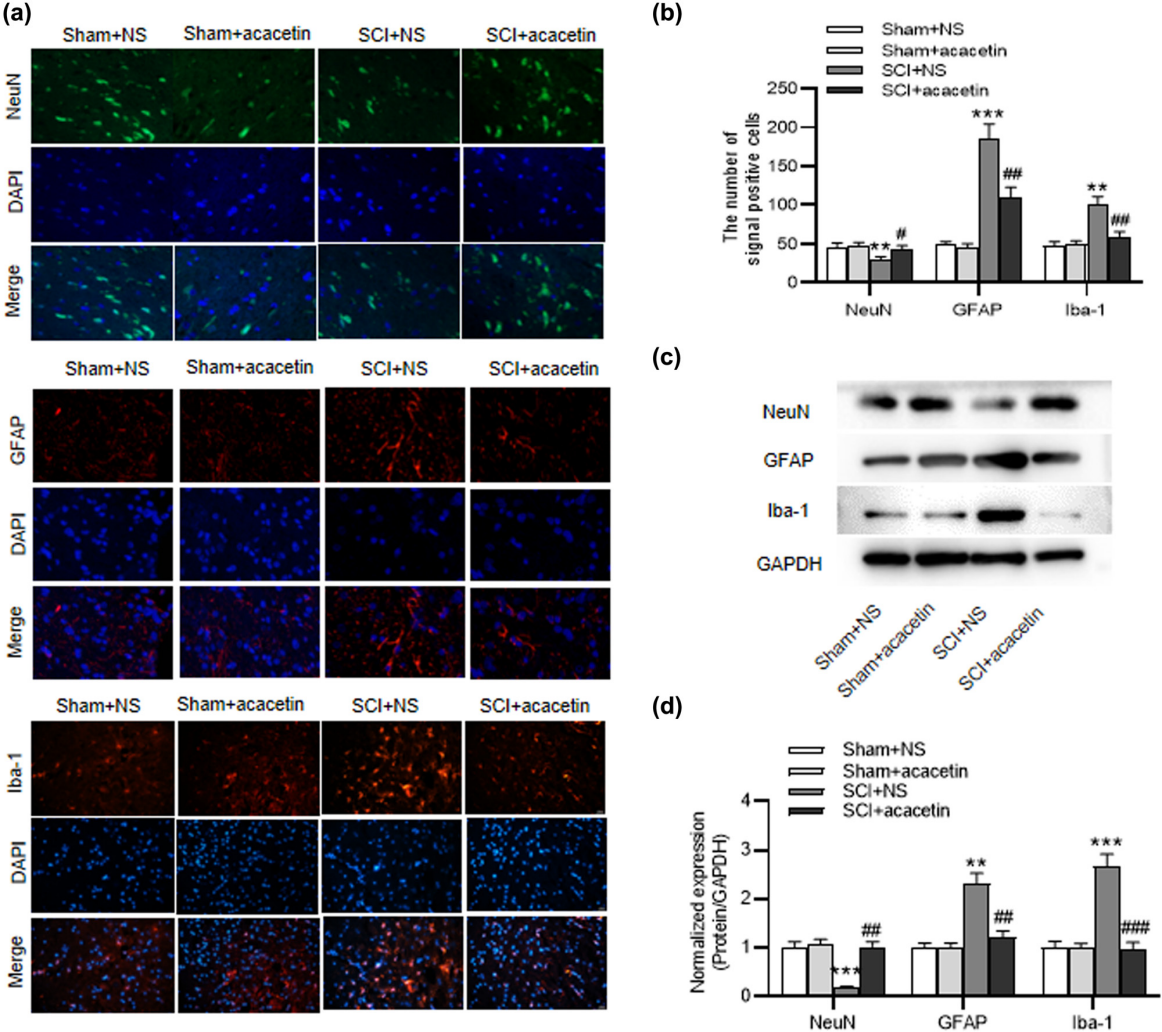
Acacetin represses glial cell activation after SCI. (a–d) Immunofluorescence and western blot were applied for detecting the levels of NeuN, GFAP, and Iba-1 in the spinal cord tissues of different groups (sham + NS group, sham + acacetin group, SCI + NS group, and SCI + acacetin group). *n* = 8 each group. **p < 0.01, ***p < 0.001 (compared with the sham + NS group); ^#^ *p* < 0.05, ^##^ *p* < 0.01, ^###^ *p* < 0.001 (compared with the SCI + NS group).

We further detected the influence of acacetin on the activation of glial cells and neuron integrity. It was illustrated from immunofluorescence assays that the quantity of NeuN-positive neurons in SCI group was lower than that in the sham-operated group, which was then recovered by acacetin treatment, suggesting that acacetin could improve neuronal survival. Moreover, SCI-stimulated increase of GFAP and Iba-1 levels were reversed by acacetin treatment, which indicated that acacetin could repress the activation of glial cell (Figure 2a and b). Similarly, the same trend was observed in western blot. The NeuN, GFAP, and Iba-1 protein levels were almost unchanged in the two sham groups. Compared with the spinal cord tissues of sham mice, NeuN protein level was significantly low in tissues of SCI mice, while acacetin treatment reversed NeuN level after SCI. The protein levels of GFAP and Iba-1 were elevated in mice after SCI, while acacetin treatment reduced their levels (Figure 2c and d).

### 3.3 Acacetin alleviates spinal cord edema of SCI mice and suppresses inflammatory response

**Figure 3:**
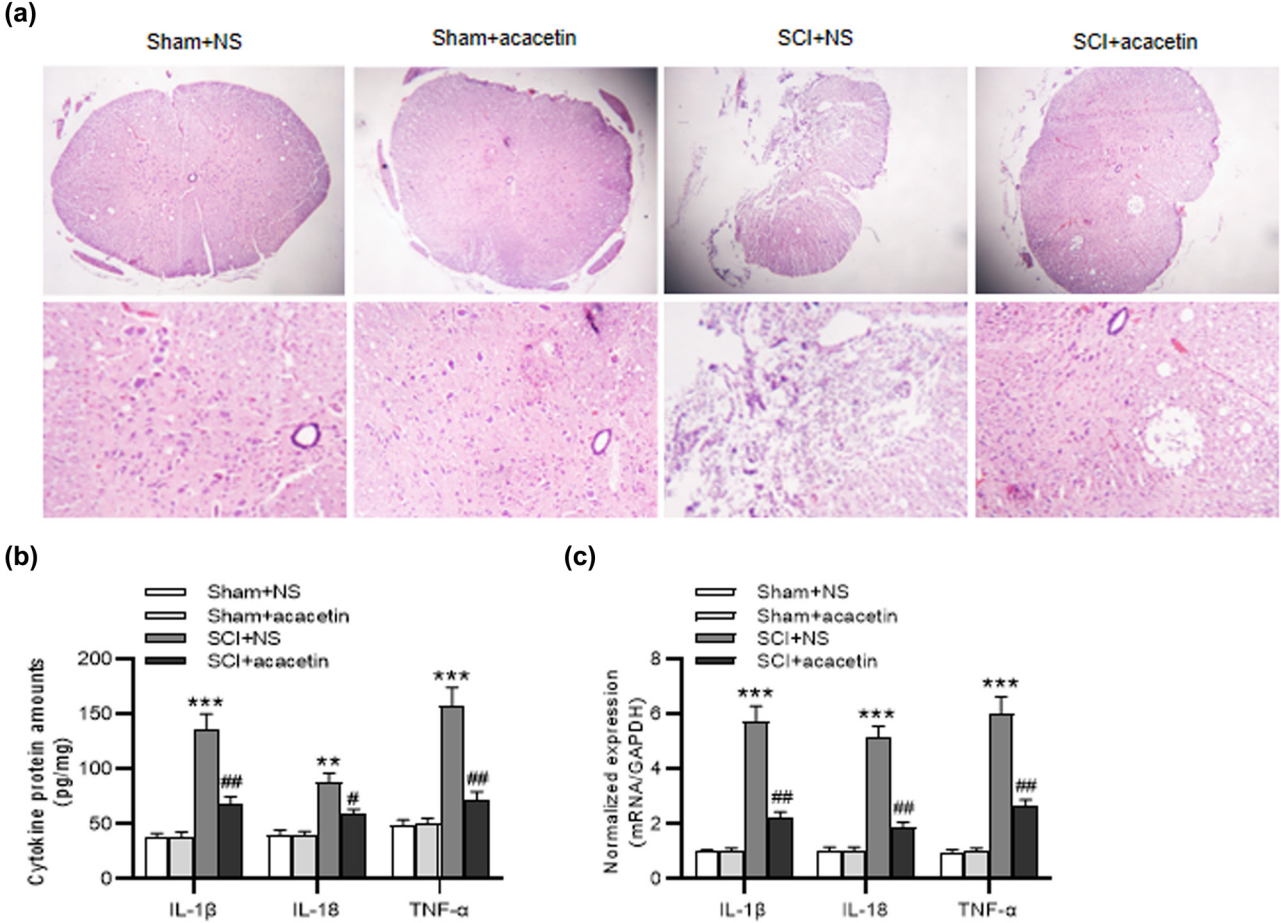
Acacetin alleviates spinal cord edema of SCI mice and suppresses inflammatory response. (a) H&E staining was utilized for testing the pathological change of the spinal cord of mice in different groups (sham + NS group, sham + acacetin group, SCI + NS group, and SCI + acacetin group). (b and c) ELISA and RT-qPCR were utilized to assess the concentrations and mRNA levels of IL-1β, IL-18, and TNF-α in the spinal cord tissues in different groups. *n* = 8 each group. **p < 0.01, ***p < 0.001 (compared with the sham + NS group); ^#^ *p* < 0.05, ^##^ *p* < 0.01 (compared with the SCI + NS group).

Next, the influence of acacetin on spinal cord edema of mice after SCI and inflammatory response was further estimated. Histopathological alterations of the spinal cord tissues were estimated by H&E staining. We found that, in the sham groups, there was no significant change in tissue morphology. We further observed obvious edema and structural injury in the spinal cord of SCI mice, while these phenomena were alleviated after acacetin treatment (Figure 3a). ELISA indicated that the concentrations of inflammatory factors (IL-1β, IL-18, and TNF-α) were almost unchanged in the sham groups, while their concentrations in SCI + NS group were elevated. However, acacetin treatment reversed this elevation (Figure 3b). RT-qPCR further indicated that IL-1β, IL-18, and TNF-α mRNA levels were decreased by acacetin post SCI (Figure 3c).

### 3.4 Acacetin relieves oxidative stress in SCI mice

**Figure 4:**
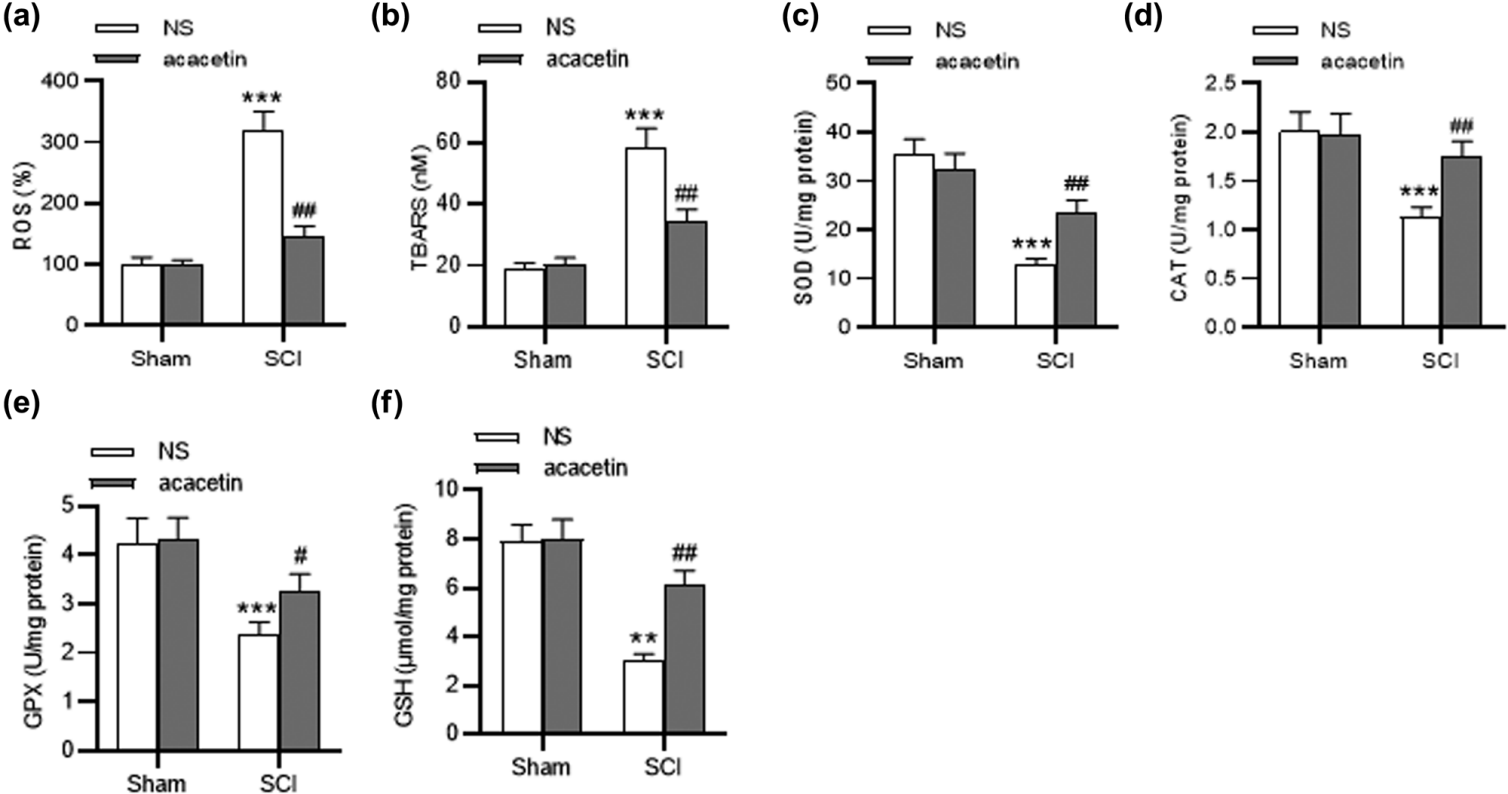
Acacetin relieves oxidative stress post SCI. (a–f) Levels of ROS, TBARS, SOD, CAT, GPX, and GSH in the spinal cord tissues in different groups (sham + NS group, sham + acacetin group, SCI + NS group, and SCI + acacetin group) were detected. *n* = 8 each group. **p < 0.01, ***p < 0.001 (compared with the sham + NS group); ^#^ *p* < 0.05, ^##^ *p* < 0.01 (compared with the SCI + NS group).

The influence of acacetin on oxidative stress in SCI mice was further investigated. We discovered that the levels of ROS and TBARS were elevated in the spinal cord tissues of SCI mice, while acacetin reversed their contents. Moreover, SCI-induced significant decrease of antioxidants (SOD, CAT, GPX, and GSH) was restored by acacetin in the spinal cord of SCI mice (Figure 4a–f).

### 3.5 Acacetin activates HO-1/Nrf2 pathway in SCI mice

**Figure 5:**
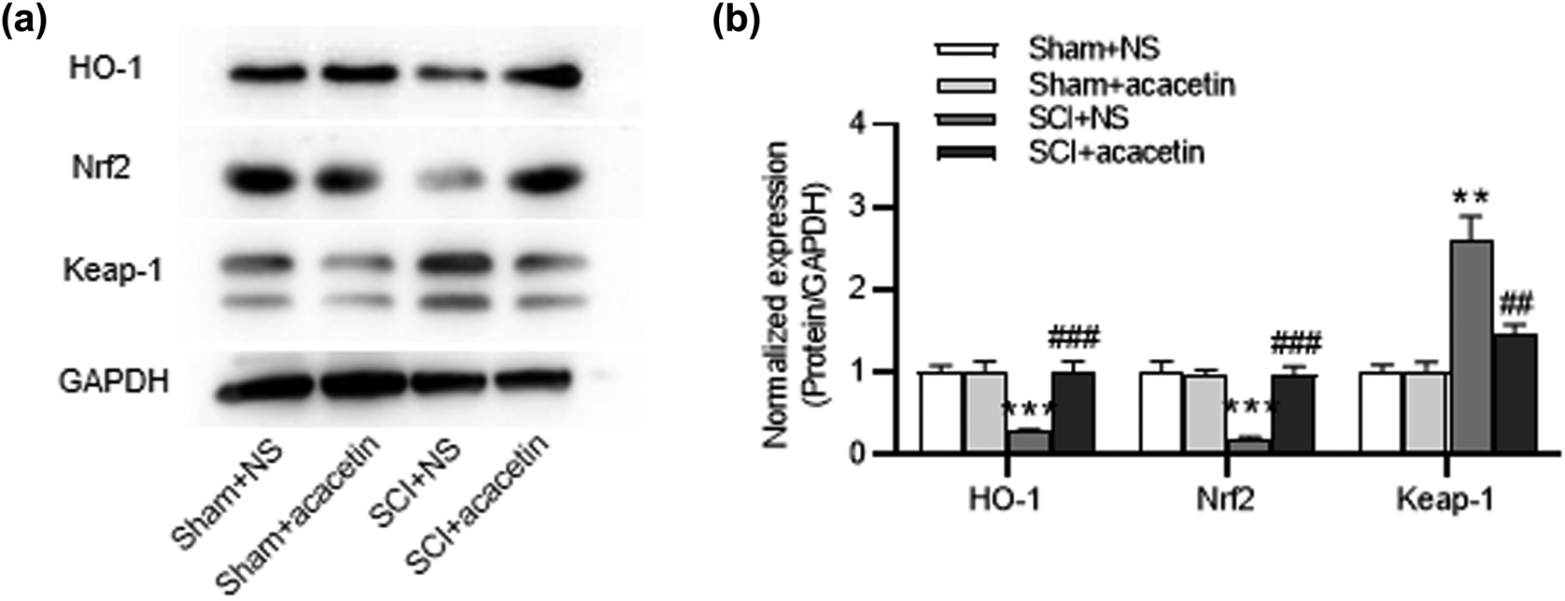
Acacetin activates HO-1/Nrf2 pathway in SCI mice. (a and b) Protein levels of HO-1, Nrf-2, and Keap-1 in the spinal cord tissues in different groups (sham + NS group, sham + acacetin group, SCI + NS group, and SCI + acacetin group) were tested by western blot. *n* = 8 each group. **p < 0.01, ***p < 0.001 (compared with the sham + NS group); ^##^ *p* < 0.01, ^###^ *p* < 0.001 (compared with the SCI + NS group).

Nrf2/HO-1 pathway has been confirmed as a crucial signaling pathway for studying oxidative stress response after SCI. Thus, we investigated whether acacetin could regulate Nrf2/HO-1 pathway in SCI mice. We discovered that the HO-1 and Nrf-2 levels were reduced in the spinal cord tissues of SCI mice in comparison to those in the sham groups, while acacetin treatment restored their levels. Instead, Keap-1 level elevated in SCI group was repressed by acacetin treatment (Figure 5a and b).

### 3.6 Acacetin exerts a neuroprotective effect by modulating the Nrf2

**Figure 6:**
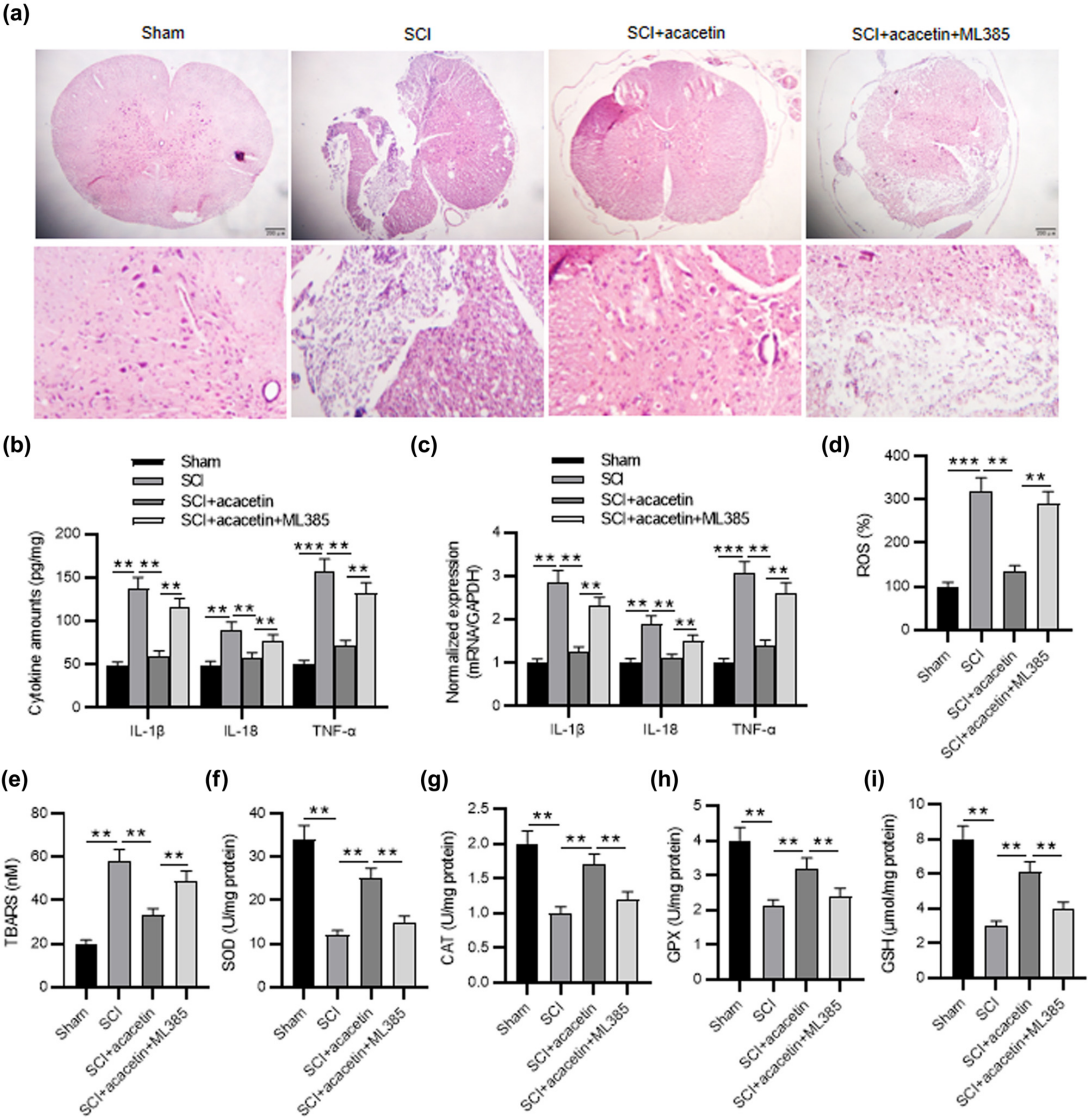
Acacetin exerts a neuroprotective effect by modulating the Nrf2. (a) H&E staining was utilized for testing the pathological change of the spinal cord of mice in different groups (sham group, SCI group, SCI + acacetin group, and SCI + acacetin + ML385 group). (b and c) ELISA and RT-qPCR were utilized to assess the concentrations and mRNA levels of IL-1β, IL-18, and TNF-α in the spinal cord tissues in different groups. (d–i) The levels of ROS, TBARS, SOD, CAT, GPX, and GSH in the spinal cord tissues in different groups. *n* = 8 each group. **p < 0.01, ***p < 0.001.

To verify the involvement of the Nrf2 signaling pathway in the neuroprotective effect of acacetin on SCI, an Nrf2 inhibitor (ML385) was used. H&E staining revealed that acacetin significantly decreased the injury of spinal cord tissue, which was abrogated by ML385 to some degree (Figure 6a). Next, we explored the effect of acacetin on SCI-induced inflammation and oxidative stress after inhibiting the Nrf2 signaling pathway. The levels of proinflammatory cytokines (TNF-α, IL-18, and IL-1β) were significantly decreased by acacetin pretreatment, and the anti-inflammatory activity of acacetin was significantly reduced by ML385 (Figure 6b and c). The SCI-mediated decrease in SOD, CAT, GPX, and GSH levels and increase in ROS and TBARS levels were attenuated by acacetin; this effect was significantly diminished by ML385 (Figure 6d–i), suggesting that acacetin protected SCI rats against oxidant stress and inflammation by activating the Nrf2 signaling pathway.

## 4 Discussion

SCI is a relatively serious type of trauma in clinic practice [1]. Secondary edema, inflammatory reaction, and oxidative stress injury after SCI can cause secondary injury to the spinal cord [36]. Therefore, it is necessary to find a treatment method that can inhibit the secondary injury after SCI. In this study, we used C57BL/6J mice to establish the SCI model. Then the locomotor function of mice was tested. It was found that SCI mice showed the impaired locomotor capability. Acacetin, a natural flavone extensively present in plant pigments, has been largely confirmed to possess assorted beneficial biological effects in different diseases [24]. For example, acacetin represses inflammatory reaction in nucleus pulposus cells and relieves intervertebral disc degeneration [37]. Acacetin ameliorates colitis in mice via inhibiting macrophage inflammatory response and regulating the composition of gut microbiota [38]. In this study, we discovered that acacetin treatment recovered the locomotor function of mice after SCI. It suggested that acacetin may exert a protective effect in SCI.

SCI is generally accompanied with the activation of glial cells [39]. The activation of microglia can lead to the production of proinflammatory factors, which can promote the neuroinflammation of SCI [14,40]. For example, estrogen alleviates neuropathic pain after SCI via suppressing microglia and astrocyte activation [41]. MiR-124-3p represses SCI by inactivating neurotoxic microglia and astrocytes [42]. Thus, we detected the influence of acacetin on neuronal apoptosis and activation of microglia via determining the expression pattern of NeuN (a marker of neurons), GFAP (a marker of astrocytes), and Iba1 (a marker of microglia) in the spinal cord tissues of SCI mice. We found that NeuN level was low in tissues of SCI mice, while acacetin treatment reversed NeuN level. The levels of GFAP and Iba-1 were elevated in mice after SCI, while acacetin treatment reduced their levels. Thus, we confirmed that acacetin could improve neuronal integrity and activation of microglia in SCI. Furthermore, acacetin treatment reduced the high levels of proinflammatory factors (IL-1β, IL-18, and TNF-α) post SCI, suggesting that the neuroinflammation could be inhibited by acacetin. Previously, it was reported that, in an LPS-induced neuroinflammation mouse model, acacetin repressed microglial activation and the levels of IL-1β and TNF-α [31]. It was consistent with our findings. Taken together, we proved that acacetin could repress neuroinflammation in SCI.

Inflammatory responses and oxidative stress are the two major types of secondary injury in SCI [43]. The above experimental results confirmed that acacetin has anti-inflammatory effects in SCI. We then explored the influence of acacetin on oxidative stress. The massive production of oxygen free radicals is the characteristic of the activation of oxidative stress response [44]. ROS is a common oxygen free radical, which can oxidize neurons in the spinal cord and causes cell damage [45]. TBARS is the product of lipid peroxidation induced by free radicals [46]. SOD, GPX, and CAT are common antioxidant enzymes and GSH is a non-enzymatic antioxidant [47]. They exert the crucial function in the balance of oxidation and antioxidation of the body [47]. It has been confirmed that they can remove free radicals and protect cells from damage [48]. Acacetin was reported to attenuate renal damage by repressing apoptosis and oxidative stress in mice [49]. Acacetin can alleviate myocardial ischemia/reperfusion injury by repressing oxidative stress and apoptosis [50]. Acacetin suppresses diabetes-induced cardiomyopathy by inhibiting oxidative stress via PPAR-α/AMPK pathway [51]. In this study, we discovered that the contents of ROS and TBARS were increased in the spinal cord tissues of SCI mice, while acacetin treatment reversed their levels. Meanwhile, acacetin treatment also reversed the decreased contents of SOD, GSH, GPX, and CAT in SCI mice. These results proved that acacetin could relieve oxidative stress injury in SCI.

Nrf2 interacts with antioxidant response elements, followed by activation of downstream antioxidant proteins [52]. Also, Nrf2 regulates the expression of a panel of antioxidants and detoxification enzymes and plays an important role in regulating oxidative stress and inflammation. Especially in CNS diseases, Nrf2 plays a key role in defending against potential oxidative stress or insults [53]. Therefore, modulating Nrf2 may prevent oxidative-stress-induced injury in CNS diseases. Nrf2 activators exert a neuroprotective effect in animal models of SCI. Zhang et al. found that sinomenine attenuated traumatic SCI by inhibiting oxidative stress and inflammation by activating the Nrf2 pathway [54]. Normally, the binding of Nrf2 to Keap-1 in the cytoplasm prevents its nuclear translocation. Stimulation by physicochemical factors disrupts this binding to Keap-1, leading to nuclear translocation of Nrf2, whereby it binds to antioxidant response elements and activates transcription of genes encoding detoxification enzymes and cytoprotective proteins, such as NQO1, SOD, and HO-1 [55]. HO-1 prevents oxidative stress, inflammation, and metabolic disorders [54,56]. The antioxidant and anti-apoptotic activities of acacetin reportedly protect against doxorubicin cardiomyopathy by upregulating Nrf2 and HO-1 expression [57]. Similarly, acacetin exerts antioxidant potential against atherosclerosis through Nrf2 pathway in apoE−/− mice [58]. To investigate whether the effect of acacetin on SCI is dependent on the presence of Nrf2, we used a Nrf2 inhibitor (ML385) in mouse SCI models. We found that ML385 reversed acacetin-mediated protective effects on SCI. This was confirmed by the alterations in the levels of SOD, CAT, GPX, GSH, ROS, TBARS, TNF-α, IL-18, and IL-1β. Therefore, acacetin exerted antioxidant and anti-inflammatory effects by activating the Nrf2 signaling pathway.

Taken together, this study confirms that acacetin alleviates neuroinflammation and oxidative stress injury via Nrf2/HO-1 signal molecules in mouse models of SCI. These findings may provide the novel therapeutic agent for SCI treatment. However, the specific molecular mechanism of how acacetin mediates the Nrf2/HO-1 signaling pathway has not been further explored, which will be the focus of our future research.
